# Differences in brain aging between sexes in Parkinson’s disease

**DOI:** 10.1038/s41531-024-00646-w

**Published:** 2024-02-14

**Authors:** Iman Beheshti, Samuel Booth, Ji Hyun Ko

**Affiliations:** 1 Department of Human Anatomy and Cell Science, University of Manitoba, Winnipeg, MB Canada; 2 Kleysen Institute for Advanced Medicine, Health Science Centre, Winnipeg, MB Canada; 3 Graduate Program in Biomedical Engineering, University of Manitoba, Winnipeg, MB Canada

**Keywords:** Parkinson's disease, Parkinson's disease, Cognitive ageing

## Abstract

Parkinson’s disease (PD) is linked to faster brain aging. Male sex is associated with higher prevalence, severe symptoms, and a faster progression rate in PD. There remains a significant gap in understanding the function of sex in the process of brain aging in PD. The structural T1-weighted MRI-driven brain-predicted age difference (i.e., Brain-PAD: the actual age subtracted from the brain-predicted age) was computed in a group of 373 people with PD (mean age ± SD: 61.37 ± 9.81, age range: 33–85, 34% female) from the Parkinson’s Progression Marker Initiative database using a robust brain-age estimation framework that was trained on 949 healthy subjects. Linear regression models were used to investigate the association between Brain-PAD and clinical variables in PD, stratified by sex. Males with Parkinson’s disease (PD-M) exhibited a significantly higher mean Brain-PAD than their female counterparts (PD-F) (*t*(256) = 2.50, *p* = 0.012). In the propensity score-matched PD-M group (PD-M*), Brain-PAD was found to be associated with a decline in general cognition, a worse degree of sleep behavior disorder, reduced visuospatial acuity, and caudate atrophy. Conversely, no significant links were observed between these factors and Brain-PAD in the PD-F group. Having ‘older’ looking brains in PD-M than PD-F supports the idea that sex plays a vital function in PD, such that the PD mechanism may be different in males and females. This study has the potential to broaden our understanding of dissimilarities in brain aging between sexes in the context of PD.

## Introduction

Parkinson’s disease (PD) is a progressive neurodegenerative disorder that is characterized by both motor and non-motor symptoms^1^, Tremors, rigidity, slowness of movement, and difficulty in walking are the most noticeable motor abnormalities found in PD^2^. In addition, the majority of people with PD (PwP) also experience cognitive decline (up to 50%), impulse control disorders (up to 60%), apathy/anhedonia (up to 40%), depression (up to 35%), and anxiety (up to 60%)^2^. A growing number of studies have attempted to identify the risk factors in PD. Although the fundamental causes of PD are yet unknown, several factors appear to play a vital role, including genetic predisposition and advanced age. Male sex is also recognized as a significant factor that contributes to the development and phenotypic expression of PD^3^. The prevalence of PD is twice as high in males compared to females and is frequently associated with earlier disease onset^4^. It has been observed that tremor-dominant PD is more prevalent in females, whereas rigid-dominant PD is more commonly found in males^4^. In the cognitive domain, male PwP are more likely to experience mild cognitive impairment and have more rapid progression to dementia^5,6^. Additionally, there are sex differences in the expression of symptoms across different cognitive domains. For example, female PwP generally perform better than males in verbal cognitive tasks^4^. Males typically experience more significant impairments in verbal fluency, verbal memory, and facial emotion identification, whereas female PD patients typically experience more diminished visuospatial cognition^3^.

These findings demonstrate that sex plays a substantial role in the clinical presentation of PD, with women displaying milder symptoms^7^. To date, a few neuroimaging studies have explored sex differences in PD. For instance, a more recent study investigated sex differences in PD using anatomical MRI data, including deformation-based morphometry, cortical thickness, and diffusion-weighted MRI measures, on a large sample of PwP^7^. Tremblay et al.^7^ reported that when disease duration and severity were equal, male PwP had more severe brain atrophy in the right postcentral gyrus, bilateral frontal lobes, left insular lobe, left thalamus, left inferior temporal gyrus, and cingulate gyrus. On the contrary, females have more severe tissue loss in the right occipital cortex, the left frontal lobe, the left insular gyrus, and the right parietal lobe^7^. Similarly, reduced cortical thickness has been observed in the frontal, temporal, occipital, and parietal lobes in male PwP than their female counterparts^8^. However, direct comparisons of brain morphology between sexes in geriatric neurodegenerative disorders may produce misleading conclusions because sex differently affects normal aging-related gray matter volume loss^9^. In addition, it is yet unknown whether the brain atrophy identified in PD is associated with “accelerated” normal aging process or a distinct pathological process apart from normal aging. For example, it has been postulated that the normal aging process is exaggerated in specific vulnerable brain regions in PD^10^, including the substantia nigra, while earlier neuroimaging studies reported a divergent whole-brain metabolic pattern in normal aging and PD^11^.

Recent advances in machine learning (ML) techniques have made it possible to estimate an individual’s “Brain Age” more accurately using their neuroimaging data^12^. The typical output of a brain age estimation model is known as Brain-PAD (i.e., brain-predicted age minus actual age), which can quantify the degree of global brain health^12^. Since Brain-PAD provides a quantitative metric that estimates the overall brain health associated with the normal aging process, it has found widespread application in different fields of neurological disorders, including Alzheimer’s disease^13^, epilepsy^14^, cocaine use disorder^15^, and schizophrenia^16^.

To date, only a few studies have investigated the brain age estimation technique in the area of PD^17,18^. These studies mostly focused on quantifying Brain-PAD and the association between Brain-PAD scores and clinical variables in PD. For instance, a strong correlation between Brain-PAD scores and Unified Parkinson’s Disease Rating Scale (UPDRS) III scores (motor symptom severity), as well as a weak correlation between Brain-PAD scores and Montreal Cognitive Assessment (MoCA) scores has been reported in PD^17,18^. However, investigation into sex differences in PD using brain age measures has not been performed. In this study, we investigated whether clinical symptom severity correlates differently with Brain-PAD in male versus female PD.

## Results

### Sex differences in clinical and biomarker scores

All diagnostic assessments and symptom ratings were obtained from the Parkinson’s Progression Markers Initiative (PPMI) (www.ppmi-info.org) dataset. The demographic characteristics, including age, education, age at diagnosis, and disease duration, were similar between female and male PD (Table 1). While the overall severity of motor symptoms (total UPDRS-III) was not significantly different between males and females (*t*(370) = 0.59, *p* = 0.551), the rigidity subscale was significantly higher in PD-M vs. PD-F (*t*(371) = 3.04, *p* = 0.002). The tremor subscale was not significantly different between sexes (*t*(371) = 0.29, *p* = 0.767).

**Table 1:** Demographics and clinical characteristics of PwP used in this study by sex.

Characteristics		PD (*N* = 373)		PD-M (*N* = 244)		PD-F (*N* = 129)		PD-M* (*N* = 129)		P*	P**
*Demographics*	#	Mean	SD	Mean	SD	Mean	SD	Mean	SD		
Age, years	0	61.37	9.81	62.04	9.87	60.11	9.63	61.14	9.93	0.07	0.40
Diagnosis age, years		60.81	9.77	61.51	9.83	59.50	9.57	60.62	9.85	0.06	0.36
Education, years	0	15.55	2.88	15.74	2.88	15.19	2.84	15.50	2.75	0.08	0.37
Disease duration, months	0	6.73	6.58	6.42	6.05	7.32	7.48	6.32	5.73	0.21	0.23
*Motor symptoms*											
UPDRS-III (total)	1	32.22	13.21	32.52	13.34	31.66	12.99	30.13	13.22	0.55	0.35
UPDRS-III (total rigidity)	0	3.83	2.63	4.13	2.67	3.26	2.46	3.63	2.62	0.00	0.25
UPDRS-III (total tremor)	0	4.34	3.16	4.37	3.18	4.27	3.13	4.28	3.17	0.77	0.98
*Non-motor symptoms*											
MoCA	0	27.07	2.32	26.88	2.34	27.43	2.25	27.10	2.32	0.03	0.24
Epworth sleepiness scale	0	5.65	3.39	5.73	3.27	5.50	3.61	5.60	3.31	0.53	0.80
Letter number sequencing score	0	10.56	2.66	10.45	2.72	10.78	2.54	10.48	2.92	0.26	0.39
REM	2	4.08	2.64	4.20	2.71	3.85	2.50	3.85	2.69	0.22	1.00
BJLO	0	12.81	2.11	13.15	2.02	12.17	2.13	12.70	2.17	0.00	0.06
HVLT delayed recall	0	8.36	2.50	8.00	2.55	9.06	2.25	8.15	2.49	0.00	0.00
HVLT delayed recognition	1	11.22	1.09	11.12	1.14	11.40	0.96	11.21	1.04	0.02	0.12
Olfactory testing	0	22.49	8.33	21.44	8.18	24.48	8.27	21.80	8.82	0.00	0.01
Symbol digit modalities score	0	41.48	9.83	40.19	9.77	43.93	9.51	40.78	9.21	0.00	0.01
*Mood*											
Anxiety	1	65.27	18.06	64.21	17.59	67.30	18.81	64.84	17.99	0.12	0.28
GDS	0	2.29	2.38	2.32	2.35	2.24	2.43	2.30	2.40	0.76	0.84
*CSF biomarkers, pg/ml*											
a-Synuclein (a-syn)	9	1502	669	1458	647	1585	702	1448	607	0.08	0.10
Amyloid-b	13	918	416	901	377	950	481	904	364	0.29	0.39
CSF p-tau (2016 assay)	39	14.78	5.19	14.61	4.73	15.10	5.97	14.43	4.88	0.42	0.35
*SPECT biomarkers*											
CAUDATE (L + R)	3	4.01	1.11	3.95	1.08	4.12	1.16	4.04	1.09	0.17	0.59
PUTAMEN (L + R)	3	1.64	0.58	1.62	0.55	1.68	0.63	1.72	0.55	0.40	0.58

*PD* Parkinson’s disease, *F* females, *M* males, M* matched males, *UPDRS* unified Parkinson’s disease rating scale, *MoCA* Montreal Cognitive Assessment, *REM* sleep behavior disorder questionnaire score, *BJLO* Benton judgment of line orientation score, *HVLT* Hopkins verbal learning test, *GDS* geriatric depression scale, *N* number of subjects, *#* number of missing values in each variable, *L* left, *R* right, P* *t*-test between PD-F and PD-M, P** *t*-test between PD-F and PD-M*. Disease duration is computed by subtracting the diagnosed age from the MRI scan age.

Significant sex differences were observed among cognitive performance scores as measured by MoCA, Benton Judgment of Line Orientation Score (BJLO), Hopkins Verbal Learning Test (HVLT) Delayed Recall, HVLT Delayed Recognition, olfactory testing, and Symbol Digit Modalities Score (*p* < 0.03). There was no significant difference between sexes in the Epworth Sleepiness Scale, Letter Number Sequencing Score, and REM (*p* > 0.2). Anxiety and Geriatric Depression Scale (GDS) scores were not significantly different between sexes (*p* > 0.1). No significant sex differences were observed in cerebrospinal fluid (CSF) biomarkers (i.e., α-Synuclein, Amyloid-β, and CSF p-tau; *p* > 0.08). Regional atrophy in caudate and putamen were not significantly different (*p* > 0.1), either.

To control for different clinical symptom severity and other demographic variables in the correlational analyses below, we used the propensity score matching method to select 129 male PD (PD-M*). We confirmed that there was no significant difference between PD-M* and PD-F in age, education level, age of diagnosis, UPDRS-III (total), UPDRS-III (total rigidity), UPDRS-III (total tremor), MoCA, Epworth sleepiness scale (ESS), letter-number sequencing, sleep behavior disorder question score (REM) (*p* > 0.2). For details, see Table 1.

### Brain age across different groups

**Fig. 1:**
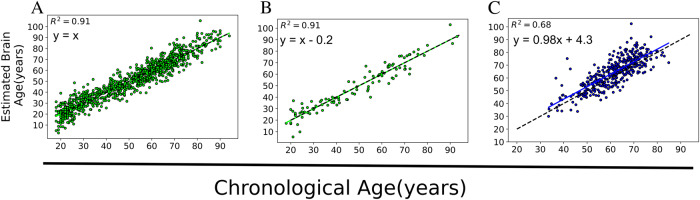
Scatter plots showing the estimated brain age versus actual age in different datasets. **A** The training set (*N* = 949) that was evaluated through a 10-fold cross-validation strategy, **B** the hold-out HC (*N* = 105), and **C** PD (*N* = 373). The dashed black line represents the identity line (*y* = *x*).

**Fig. 2:**
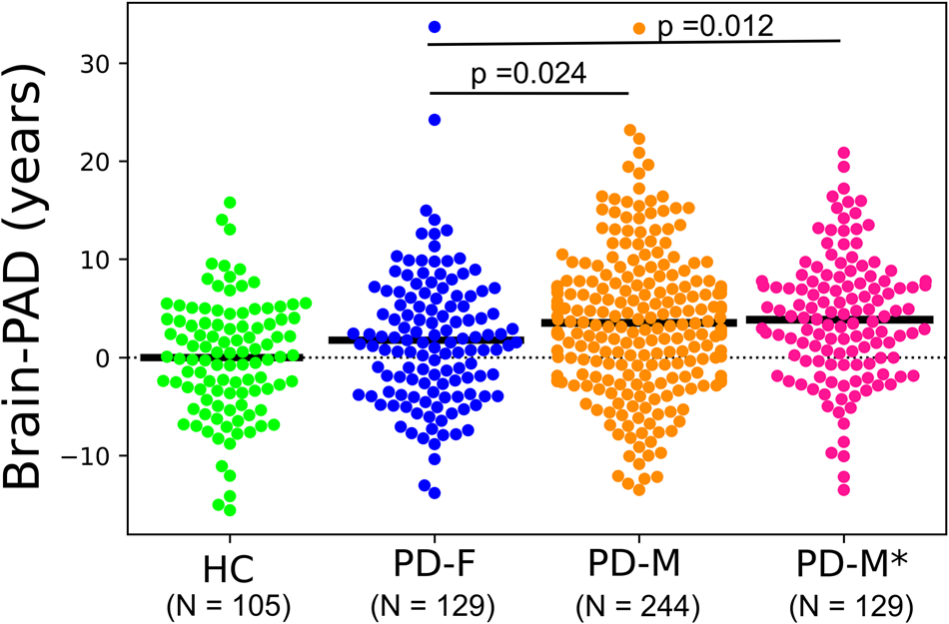
Box-plots showing the grouped Brain-PAD values among the hold-out HC and PwP with respect to the sex categories. The solid black line represents the mean Brain-PAD values of each group, while the dashed black line represents the reference line (*y* = 0). The statistical tests between groups were performed using a student *t*-test.

The mean absolute error (MAE) in the training set (achieved through a 10-fold cross-validation strategy) and the hold-out healthy controls (HC) set were 4.72 years and 4.63 years, respectively. The mean of Brain-PAD in the training and hold-out HC sets was approximately zero (Table 2). There was no significant difference between males and females in the training set (*t*(947) = 0.52, *p* = 0.60) as well as the hold-out HC (*t*(103) = 0.56, *p* = 0.57) in Brain-PAD. As expected, the PwP showed a positive mean Brain-PAD of 2.92 years, which was significantly higher than that of the hold-out HC (*t*(476) = 3.94, *p* < 0.001). Our mean Brain-PAD of 2.92 years is similar to what has been reported in PD^17,18^. Figure 1 displays the association between estimated ages versus the actual ages in different groups. Regression parameters were almost identical between training and hold-out sets of HCs, while PwP showed higher intersection (+4.3 years) than the identity line (*t*(140) = 5.00, *p* < 0.001) and increased variability reflected by the decreased R2 (0.63–0.73 vs. 0.91). Figure 2 shows the contrasts of Brain-PAD in different subsets. All PD subgroups showed higher Brain-PAD than HC (hold-out set) (*p* < 0.05). PD-M group had a significantly higher Brain-PAD than PD-F group (*t*(371) = 2.26, *p* = 0.024) (Table 2). The significance was preserved when PD-M* was compared with PD-F (*t*(256) = 2.50, *P* = 0.012).

**Table 2:** The results of brain age values on different sets.

Set	Group	*N*	MAE (y)	RMSE (y)	R2	Mean Brain-PAD (y)	95% CI values (y)
Training set	HC	949	4.72	6.07	0.91	0.00	−0.38, 0.38
Hold-out set	HC	105	4.63	5.88	0.91	-0.08	−1.23, 1.05
Test set	PD (all)	373	5.96	7.72	0.68	2.92	2.20, 3.65
Test set	PD-F	129	5.30	7.00	0.63	1.78	0.59, 2.96
Test set	PD-M	244	6.31	8.08	0.64	3.53	2.61, 4.45
Test set	PD-M*	129	6.02	7.49	0.73	3.85	2.72, 4.95

### Brain age and motor symptoms

Multiple linear regression models for predicting UPDRS-III (total) score were significant in PD (all), PD-M, and PD-M* (*p* < 0.01, FDR corrected) but not in PD-F (*p* = 0.308). In PD, increased UPDRS-III scores were associated with higher Brain-PAD (*t*(366) = 2.81, *p* = 0.005), actual age (*t*(366) = 3.33, *p* = 0.001), and duration of the disease (*t*(366) = 2.56, *p* = 0.011) (Table 3). This pattern was similar in the PD-M group, but an increase in UPDRS-III (total) scores in the PD-M* group was associated only with a higher actual age (*t*(124) = 2.61, *p* = 0.010). No association was observed between Brain-PAD and the UPDRS-III (total rigidity) or between Brain-PAD and the UPDRS-III (total tremor) in our investigation.

**Table 3:** Regression Model Outputs in PD: Motor Symptoms.

Model		UPDRS-III (total)				UPDRS-III (total rigidity)				UPDRS-III (total tremor)			
		PD	PD-F	PD-M	PD-M*	PD	PD-F	PD-M	PD-M*	PD	PD-F	PD-M	PD-M*
*R*^2^		0.067	0.040	0.114	0.111	0.035	0.039	0.013	0.015	0.063	0.067	0.066	0.094
*F* (*p*-value^†^)		4.376 **(<0.001)**	1.283 (0.308)	7.713 **(<0.001)**	3.856 **(0.008)**	2.243 **(0.049)**	1.242 (0.321)	0.763 (0.572)	0.469 (0.759)	4.134 **(0.001)**	2.242 (0.083)	4.221 **(0.004)**	3.236 **(0.020)**
Brain-PAD	b	0.265	0.159	0.295	0.182	0.031	0.020	0.033	0.007	-0.016	-0.056	0.001	0.022
	t	2.815	0.939	2.641	1.047	1.640	0.637	1.414	0.181	-0.712	-1.380	0.031	0.510
	p	**0.005**	0.350	**0.009**	0.297	0.102	0.525	0.159	0.857	0.477	0.170	0.976	0.611
Actual age	b	0.230	-0.029	0.341	0.302	0.002	-0.024	0.014	0.011	0.058	0.038	0.068	0.069
	t	3.337	-0.238	4.112	2.617	0.151	-1.052	0.808	0.454	3.545	1.337	3.349	2.450
	p	**0.001**	0.812	**<0.001**	**0.010**	0.880	0.295	0.420	0.651	**<0.001**	0.184	**0.001**	**0.016**
Duration	b	0.264	0.232	0.283	0.295	0.025	0.057	-0.001	0.023	0.057	0.057	0.060	0.097
	t	2.565	1.482	2.090	1.464	1.189	1.967	-0.045	0.536	2.321	1.542	1.796	1.990
	p	**0.011**	0.141	**0.038**	0.146	0.235	0.051	0.964	0.593	**0.021**	0.126	0.074	**0.049**
Education	b	0.007	-0.584	0.250	0.540	0.019	-0.010	0.032	0.084	0.082	0.135	0.050	-0.007
	t	0.030	-1.446	0.884	1.310	0.408	-0.130	0.542	0.975	1.469	1.406	0.727	-0.067
	p	0.976	0.151	0.377	0.193	0.683	0.897	0.588	0.331	0.143	0.162	0.468	0.947
Sex	b	-0.121	-	-	-	-0.816	-	-	-	-0.023	-	-	-
	t	-0.085	-	-	-	-2.833	-	-	-	-0.068	-	-	-
	p	0.932	-	-	-	**0.005**	-	-	-	0.946	-	-	-

*PD* Parkinson’s disease, *F* females, *M* males, M* matched males, *UPDRS* Unified Parkinson Disease Rating Scale. Significant results are highlighted in bold. ^†^The *p*-values of the *F*-statistics were adjusted for multiple comparisons using the FDR technique.

### Brain age and cognitive symptoms

The most tested multiple linear regression models were significant when predicting cognitive measures in PD (Table 4). As expected, lower MoCA scores in PD were associated with a higher Actual age (*t*(365) = −3.78, *p* < 0.001) and lower education (*t*(365) = 2.05, *p* = 0.041). In PD-M, these associations were generally maintained while higher Brain-PAD was additionally correlated with lower MoCA (*t*(238) = −2.01, *p* = 0.045). The association between MoCA and Brain-PAD remained to be significant in the PD-M* group (*t*(123) = −2.14, *p* = 0.034), while the model was not significant for predicting MoCA in PD-F group (*p* = 0.343).

**Table 4:** Regression Model Outputs in PD: Cognitive Symptoms.

Model		MoCA				Epworth sleepiness scale score				Letter Number Sequencing Score			
		PD	PD-F	PD-M	PD-M*	PD	PD-F	PD-M	PD-M*	PD	PD-F	PD-M	PD-M*
*R*^2^		0.089	0.046	0.103	0.127	0.063	0.071	0.094	0.085	0.176	0.144	0.205	0.213
*F* (*p*-value^†^)		5.081 **(<0.001)**	1.183 (0.343)	5.485 **(<0.001)**	3.564 **(0.008)**	3.484 **(0.002)**	1.854 (0.129)	4.952 **(<0.001)**	2.288 (0.062)	11.138 **(<0.001)**	4.119 **(0.003)**	12.306 **(<0.001)**	6.646 **(<0.001)**
Brain-PAD	b	-0.030	-0.008	-0.040	-0.065	0.007	-0.036	0.028	0.062	-0.037	-0.042	-0.033	-0.065
	t	-1.818	-0.278	-2.016	-2.143	0.279	-0.767	1.001	1.385	-2.074	-1.310	-1.508	-1.769
	p	0.070	0.782	**0.045**	**0.034**	0.780	0.444	0.318	0.168	**0.039**	0.193	0.133	0.079
Actual age	b	-0.046	-0.026	-0.058	-0.005	-0.005	-0.035	0.013	0.007	-0.100	-0.065	-0.114	-0.115
	t	-3.787	-1.232	-3.830	-0.258	-0.262	-1.054	0.595	0.247	-7.516	-2.853	-6.880	-4.647
	p	**<0.001**	0.220	**<0.001**	0.797	0.793	0.294	0.552	0.805	**<0.001**	**0.005**	**<0.001**	**<0.001**
Duration	b	-0.017	-0.005	-0.027	-0.103	0.045	-0.029	0.105	0.120	0.039	0.027	0.047	0.044
	t	-0.922	-0.176	-1.123	-2.901	1.668	-0.671	3.090	2.315	1.978	0.936	1.781	1.046
	p	0.357	0.861	0.262	**0.004**	0.096	0.504	**0.002**	0.022	**0.049**	0.351	0.076	0.298
Education	b	0.083	0.056	0.098	0.084	0.011	0.069	-0.029	-0.006	0.147	0.239	0.111	0.061
	t	2.052	0.788	1.957	1.153	0.178	0.617	-0.406	-0.056	3.317	3.151	2.022	0.704
	p	**0.041**	0.432	0.052	0.251	0.859	0.539	0.685	0.956	**0.001**	**0.002**	**0.044**	0.482
Sex	b	0.436	-	-	-	-0.185	-	-	-	0.091	-	-	-
	t	1.758	-	-	-	-0.502	-	-	-	0.337	-	-	-
	p	0.080	-	-	-	0.616	-	-	-	0.736	-	-	-
UPDRS III	b	-0.017	-0.027	-0.009	-0.020	0.056	0.068	0.046	0.028	-0.009	0.005	-0.012	-0.023
	t	-1.853	-1.713	-0.825	-1.256	4.163	2.757	2.851	1.242	-0.916	0.321	-0.936	-1.243
	p	0.065	0.089	0.410	0.212	**<0.001**	0.007	**0.005**	0.217	0.360	0.749	0.350	0.216

Model		Sleep Behavior Disorder Questionnaire Score (REM)				Benton Judgement of Line Orientation Score (BJLO)				HVLT Delayed Recall			
		PD	PD-F	PD-M	PD-M*	PD	PD-F	PD-M	PD-M*	PD	PD-F	PD-M	PD-M*
*R*^2^		0.132	0.174	0.137	0.213	0.152	0.127	0.108	0.151	0.195	0.167	0.161	0.147
*F* (*p*-value^†^)		7.721 **(<0.001)**	4.948 **(<0.001)**	7.555 **(<0.001)**	6.607 **(<0.001)**	9.331 **(<0.001)**	3.553 **(0.008)**	5.751 **(<0.001)**	4.392 **(0.002)**	12.582 **(<0.001)**	4.989 **(<0.001)**	9.058 **(<0.001)**	4.254 **(0.002)**
Brain-PAD	b	0.057	0.027	0.072	0.119	-0.034	-0.041	-0.030	-0.061	-0.002	0.007	-0.006	-0.016
	t	3.097	0.884	3.153	3.536	-2.365	-1.570	-1.764	-2.175	-0.107	0.247	-0.298	-0.490
	p	**0.002**	0.378	**0.002**	**0.001**	**0.019**	0.119	0.079	**0.032**	0.915	0.805	0.766	0.625
Actual age	b	-0.018	-0.043	-0.007	-0.024	-0.032	-0.047	-0.024	-0.010	-0.080	-0.076	-0.083	-0.067
	t	-1.358	-1.947	-0.414	-1.072	-3.047	-2.495	-1.818	-0.540	-6.502	-3.854	-5.198	-3.054
	p	0.175	0.054	0.679	0.286	**0.002**	**0.014**	0.070	0.590	**<0.001**	**<0.001**	**<0.001**	**0.003**
Duration	b	-0.006	-0.063	0.040	0.095	-0.002	-0.011	0.005	<0.001	0.004	0.018	-0.007	-0.027
	t	-0.307	-2.227	1.465	2.422	-0.147	-0.438	0.233	0.001	0.230	0.700	-0.281	-0.717
	p	0.759	0.028	0.144	0.017	0.883	0.662	0.816	0.999	0.818	0.485	0.779	0.474
Education	b	-0.017	-0.045	-0.015	-0.020	0.170	0.166	0.171	0.188	0.200	0.165	0.218	0.245
	t	-0.378	-0.610	-0.260	-0.254	4.839	2.635	3.967	2.822	4.880	2.505	4.127	3.176
	p	0.705	0.543	0.795	0.800	**<0.001**	**0.010**	**<0.001**	**0.006**	**<0.001**	**0.014**	**<0.001**	**0.002**
Sex	b	-0.242	-	-	-	-0.994	-	-	-	1.003	-	-	-
	t	-0.873	-	-	-	-4.602	-	-	-	3.993	-	-	-
	p	0.383	-	-	-	<0.001	-	-	-	**<0.001**	-	-	-
UPDRS-III	b	0.060	0.060	0.056	0.056	-0.017	-0.012	-0.021	-0.040	-0.009	-0.015	-0.007	-0.004
	t	5.953	3.715	4.319	3.225	-2.119	-0.833	-2.174	-2.795	-1.021	-1.001	-0.543	-0.231
	p	**<0.001**	**<0.001**	**<0.001**	**0.002**	**0.035**	0.407	**0.031**	**0.006**	0.308	0.319	0.587	0.818

Model		HVLT Delayed Recognition				olfactory testing				Symbol Digit Modalities Score			
		PD	PD-F	PD-M	PD-M*	PD	PD-F	PD-M	PD-M*	PD	PD-F	PD-M	PD-M*
*R*^2^		0.134	0.118	0.134	0.19	0.147	0.117	0.123	0.127	0.301	0.225	0.311	0.333
*F* (*p*-value^†^)		7.998 **(<0.001)**	3.253 **(0.013)**	7.365 **(<0.001)**	5.769 **(<0.001)**	8.959 **(<0.001)**	3.228 **(0.013)**	6.693 **(<0.001)**	3.564 **(0.008)**	22.377 **(<0.001)**	7.093 **(<0.001)**	21.526 **(<0.001)**	12.298 **(<0.001)**
Brain-PAD	b	0.001	-0.005	0.003	-0.006	-0.047	0.034	-0.086	0.024	-0.092	-0.181	-0.044	-0.055
	t	0.077	-0.404	0.310	-0.438	-0.820	0.326	-1.236	0.204	-1.498	-1.624	-0.598	-0.517
	p	0.939	0.687	0.757	0.662	0.413	0.745	0.218	0.839	0.135	0.107	0.550	0.606
Actual age	b	-0.029	-0.021	-0.034	-0.023	-0.266	-0.279	-0.253	-0.279	-0.398	-0.396	-0.386	-0.340
	t	-5.233	-2.370	-4.657	-2.562	-6.285	-3.713	-4.829	-3.543	-8.823	-4.948	-6.947	-4.729
	p	**<0.001**	**0.019**	**<0.001**	**0.012**	**<0.001**	**<0.001**	**<0.001**	**0.001**	**<0.001**	**<0.001**	**<0.001**	**<0.001**
Duration	b	0.008	0.023	-0.005	-0.012	0.057	0.078	0.041	-0.038	0.002	0.020	-0.015	-0.086
	t	0.924	2.059	-0.426	-0.785	0.905	0.802	0.493	-0.282	0.036	0.192	-0.164	-0.700
	p	0.356	**0.042**	0.671	0.434	0.366	0.424	0.623	0.779	0.972	0.848	0.870	0.486
Education	b	0.062	0.059	0.068	0.131	0.077	0.190	0.020	-0.014	0.713	0.666	0.781	0.861
	t	3.354	2.033	2.816	4.203	0.548	0.760	0.116	-0.050	4.751	2.502	4.259	3.420
	p	**0.001**	**0.044**	**0.005**	**<0.001**	0.584	0.449	0.907	0.960	**<0.001**	**0.014**	**<0.001**	**0.001**
Sex	b	0.256	-	-	-	2.392	-	-	-	3.159	-	-	-
	t	2.251	-	-	-	2.770	-	-	-	3.434	-	-	-
	p	**0.025**	**-**	**-**	**-**	**0.006**	**-**	**-**	**-**	**0.001**	-	-	-
UPDRS-III	b	-0.010	-0.010	-0.009	-0.010	-0.055	-0.047	-0.057	-0.062	-0.145	-0.059	-0.194	-0.208
	t	-2.364	-1.551	-1.610	-1.537	-1.736	-0.845	-1.444	-1.039	-4.308	-1.000	-4.629	-3.820
	p	**0.019**	0.124	0.109	0.127	0.083	0.400	0.150	0.301	**<0.001**	0.319	**<0.001**	**<0.001**

*PD* Parkinson’s disease, *F* females, *M* males, M* matched males, *MoCA* Montreal Cognitive Assessment, *REM* Sleep Behavior Disorder Questionnaire Score, *BJLO* Benton Judgment of Line Orientation Score, *HVLT* Hopkins Verbal Learning Test. Significant results are highlighted in bold. ^†^ The *p*-values of the *F*-statistics were adjusted for multiple comparisons using the FDR technique.

Among the rest of the cognitive test batteries, Brain-PAD was associated with the letter number sequencing score (*t*(365) = −2.074, *p* = 0.039), REM (*t*(363) = 3.09, *p* = 0.002), and BJLO scores in PD (*t*(365) = −2.36, *p* = 0.019). None of these were significant when evaluated within PD-F (*p* > 0.1), while REM was significantly correlated with Brain-PAD within both PD-M (*t*(237) = 3.15, *p* = 0.002) and PD-M* (*t*(122) = 3.53, *p* = 0.001). Only a trend-level of association was noted between Brain-PAD and BJLO scores in PD-M (*t*(238) = −1.76, *p* = 0.079), which was significant within PD-M* (*t*(123) = −2.17, *p* = 0.032). Brain-PAD was not significantly associated with HVLT delayed recall scores, HVLT delayed recognition, olfactory testing, and symbol digit modalities scores. Instead, actual age was correlated with HVLT Delayed Recall scores, HVLT Delayed Recognition, olfactory testing, and symbol digit modalities scores in all subgroups (*p* < 0.02). Details of these multiple linear regression analyses are depicted in Table 4.

### Brain age, mood, and anxiety

While Brain-PAD was not associated with anxiety or GDS scores, these symptom scores were associated with increased MoCA scores in all groups (*p* < 0.001; Table 5). Anxiety scores were negatively associated with Actual age in PD (*t*(364) = −3.79, *p* < 0.001), PD-M (*t*(237) = −3.76, *p* < 0.001), and PD-M* (*t*(122) = −2.12, *p* = 0.036), but not in PD-F (*t*(121) = −1.38, *p* = 0.17). In addition, anxiety was associated with the duration of the disease (*t*(364) = −2.09, *p* = 0.037) and level of education (*t*(364) = −2.51, *p* = 0.012) in the PD group.

**Table 5:** Regression model outputs in PD: mood and anxiety.

Model		Anxiety				GDS			
		PD	PD-F	PD-M	PD-M*	PD	PD-F	PD-M	PD-M*
*R*^2^		0.140	0.192	0.129	0.124	0.121	0.203	0.103	0.118
*F* (*p*-value^†^)		7.412 **(**<**0.001)**	4.898 **(**<**0.001)**	5.829 **(**<**0.001)**	2.876 **(0.017)**	6.231 **(**<**0.001)**	5.149 **(**<**0.001)**	4.550 **(**<**0.001)**	2.725 **(0.021)**
Brain-PAD	b	0.132	0.282	0.063	−0.054	0.004	−0.026	0.018	<0.001
	t	1.053	1.235	0.422	−0.221	0.225	−0.876	0.902	−0.008
	p	0.293	0.219	0.673	0.825	0.822	0.383	0.368	0.994
Actual age	b	−0.356	−0.228	−0.437	−0.343	−0.030	−0.040	−0.024	−0.012
	t	−3.791	−1.381	−3.765	−2.122	−2.418	−1.884	−1.555	−0.568
	p	**<0.001**	0.170	**<0.001**	**0.036**	**0.016**	0.062	0.121	0.571
Duration	b	−0.286	−0.607	−0.051	0.074	−0.005	−0.052	0.031	0.060
	t	−2.091	−2.873	−0.284	0.260	−0.291	−1.892	1.281	1.562
	p	**0.037**	**0.005**	0.777	0.796	0.771	0.061	0.201	0.121
Education	b	−0.776	−1.080	−0.616	−0.492	−0.100	−0.099	−0.102	−0.047
	t	−2.517	−1.977	−1.642	−0.866	−2.443	−1.412	−2.016	−0.611
	p	**0.012**	0.050	0.102	0.388	**0.015**	0.161	**0.045**	0.542
Sex	b	2.899	–	–	–	−0.135	–	–	–
	t	1.536	–	–	–	−0.538	–	–	–
	p	0.125	–	–	–	0.591	–	–	–
MoCA	b	0.390	0.413	0.389	0.440	0.057	0.072	0.048	0.043
	t	5.643	3.377	4.568	3.571	6.236	4.572	4.195	2.607
	p	**<0.001**	**0.001**	**<0.001**	**0.001**	**<0.001**	**<0.001**	**<0.001**	**0.010**
UPDRS-III	b	−0.333	0.492	−0.778	−0.706	<0.001	0.090	−0.031	−0.141
	t	−0.840	0.705	−1.614	−1.003	−0.004	0.999	−0.472	−1.499
	p	0.402	0.482	0.108	0.318	0.997	0.320	0.637	0.136

*PD* Parkinson’s disease, *F* females, *M* males, M* matched males, *GDS* geriatric depression scale. Significant results are highlighted in bold. ^†^The *p*-values of the *F*-statistics were adjusted for multiple comparisons using the FDR technique.

### CSF and SPECT biomarkers in PD

Prediction models for a-syn and amyloid-b levels were significant in PD (*p* < 0.02) as well as PD-F (*p* < 0.04), where Brain-PAD was always a significant predictor (*p* < 0.002) (Table 6). However, the models became non-significant when analyzed within PD-M or PD-M* (*p* > 0.1). The CSF p-tau level was not correlated with Brain-PAD, but it was correlated with actual age in PD, PD-M, and PD-F (*p* < 0.005) (Table 6). Among DaTScan single-photon emission computed tomography (SPECT) measurements, prediction models were significant in PD and PD-M (*p* < 0.04), but not in PD-F and PD-M* (*p* > 0.05) (Table 7). Brain-PAD was not associated with caudate or putamen DaTScan measures, while actual age was a significant predictor in PD and PD-M (*p* < 0.03).

**Table 6:** Regression model outputs in PD: CSF biomarkers (pg/mL).

Model		a-Synuclein (a-syn)				Amyloid-b				CSF p-tau (2016 assay)			
		PD	PD-F	PD-M	PD-M*	PD	PD-F	PD-M	PD-M*	PD	PD-F	PD-M	PD-M*
*R*^2^		0.092	0.234	0.033	0.034	0.045	0.089	0.028	0.020	0.08	0.11	0.07	0.06
*F* (*p*-value^†^)		6.091 **(**<**0.001)**	9.350 **(**<**0.001)**	1.540 (0.219)	1.062 (0.399)	2.746 **(0.018)**	2.882 **(0.034)**	1.645 (0.191)	0.612 (0.663)	4.824 **(**<**0.001)**	3.603 **(0.013)**	4.085 **(0.005)**	1.668 (0.191)
Brain-PAD	b	−15.45	−32.68	−8.89	−10.69	−10.39	−19.479	−6.897	−7.799	−0.050	−0.160	−0.015	0.049
	t	−3.117	−3.644	−1.517	−1.251	−3.280	−2.890	−2.010	−1.505	−1.206	−1.753	−0.328	0.707
	p	**0.002**	**<0.001**	0.131	0.213	**0.001**	**0.005**	0.046	0.135	0.229	0.082	0.743	0.481
Actual age	b	10.51	15.43	8.042	5.669	0.577	0.849	0.651	−0.097	0.135	0.168	0.122	0.101
	t	2.988	2.624	1.866	0.977	0.256	0.190	0.259	−0.028	4.684	2.893	3.837	2.126
	p	**0.003**	**0.010**	0.063	0.331	0.798	0.850	0.796	0.978	**<0.001**	**0.005**	**<0.001**	0.035
Duration	b	12.13	27.21	0.624	11.672	0.944	9.153	−4.865	−1.162	0.024	0.106	−0.047	0.058
	t	2.330	3.580	0.090	1.238	0.283	1.583	−1.204	−0.203	0.565	1.501	−0.903	0.523
	p	**0.020**	**<0.001**	0.928	0.218	0.777	0.116	0.230	0.839	0.573	0.136	0.368	0.601
Education	b	−3.129	4.472	−6.392	−13.122	−4.139	5.271	−9.018	−5.135	0.067	0.050	0.080	0.110
	t	−0.262	0.227	−0.433	−0.697	−0.544	0.355	−1.051	−0.452	0.696	0.269	0.736	0.676
	p	0.794	0.821	0.666	0.487	0.587	0.723	0.294	0.652	0.487	0.789	0.462	0.500
Sex	b	106.4	–	–	–	26.29	–	–	–	0.543	–	–	–
	t	1.466	–	–	–	0.564	–	–	–	0.922	–	–	–
	p	0.144	–	–	–	0.573	–	–	–	0.357	–	–	–

*PD* Parkinson’s disease, *F* females, *M* males, M* matched males. Significant results are highlighted in bold. ^†^The *p*-values of the *F*-statistics were adjusted for multiple comparisons using the FDR technique.

**Table 7:** Regression model outputs in PD: SPECT biomarkers.

Model		Caudate (L + R)				Putamen (L + R)			
		PD	PD-F	PD-M	PD-M*	PD	PD-F	PD-M	PD-M*
*R*^2^		0.052	0.040	0.052	0.076	0.043	0.045	0.044	0.021
*F* (*p*-value^†^)		3.309 **(0.006)**	1.291 (0.308)	3.244 **(0.018)**	2.546 (0.054)	2.750 **(0.018)**	1.458 (0.248)	2.736 **(0.038)**	0.647 (0.646)
Brain-PAD	b	−0.008	0.008	−0.015	−0.031	−0.004	0.006	−0.008	−0.006
	t	−0.956	0.543	−1.603	−2.013	−0.834	0.741	−1.587	−0.814
	p	0.340	0.588	0.110	0.016	0.405	0.460	0.114	0.417
Actual age	b	−0.023	−0.023	−0.022	−0.018	−0.007	−0.004	−0.008	−0.005
	t	−3.892	−2.117	−3.221	−1.786	−2.332	−0.721	−2.321	−0.946
	p	**<0.001**	0.036	**0.001**	0.077	**0.020**	0.472	**0.021**	0.346
Duration	b	0.001	<0.001	<0.001	0.005	−0.010	−0.014	−0.006	−0.003
	t	0.066	0.034	0.013	0.268	−2.126	−1.934	−1.040	−0.295
	p	0.947	0.973	0.990	0.789	0.034	0.055	0.299	0.769
Education	b	0.010	−0.003	0.017	0.008	−0.013	−0.013	−0.014	−0.017
	t	0.526	−0.079	0.726	0.229	−1.264	−0.641	−1.116	−0.982
	p	0.600	0.937	0.469	0.819	0.207	0.523	0.265	0.328
Sex	b	0.116	–	–	–	0.035	–	–	–
	t	0.956	–	–	–	0.544	–	–	–
	p	0.340	–	–	–	0.587	–	–	–

*PD* Parkinson’s disease, *F* females, *M* males, M* matched males. Significant results are highlighted in bold. ^†^The *p*-values of the *F*-statistics were adjusted for multiple comparisons using the FDR technique.

### Association between GM/WM changes and Brain-PAD

**Fig. 3:**
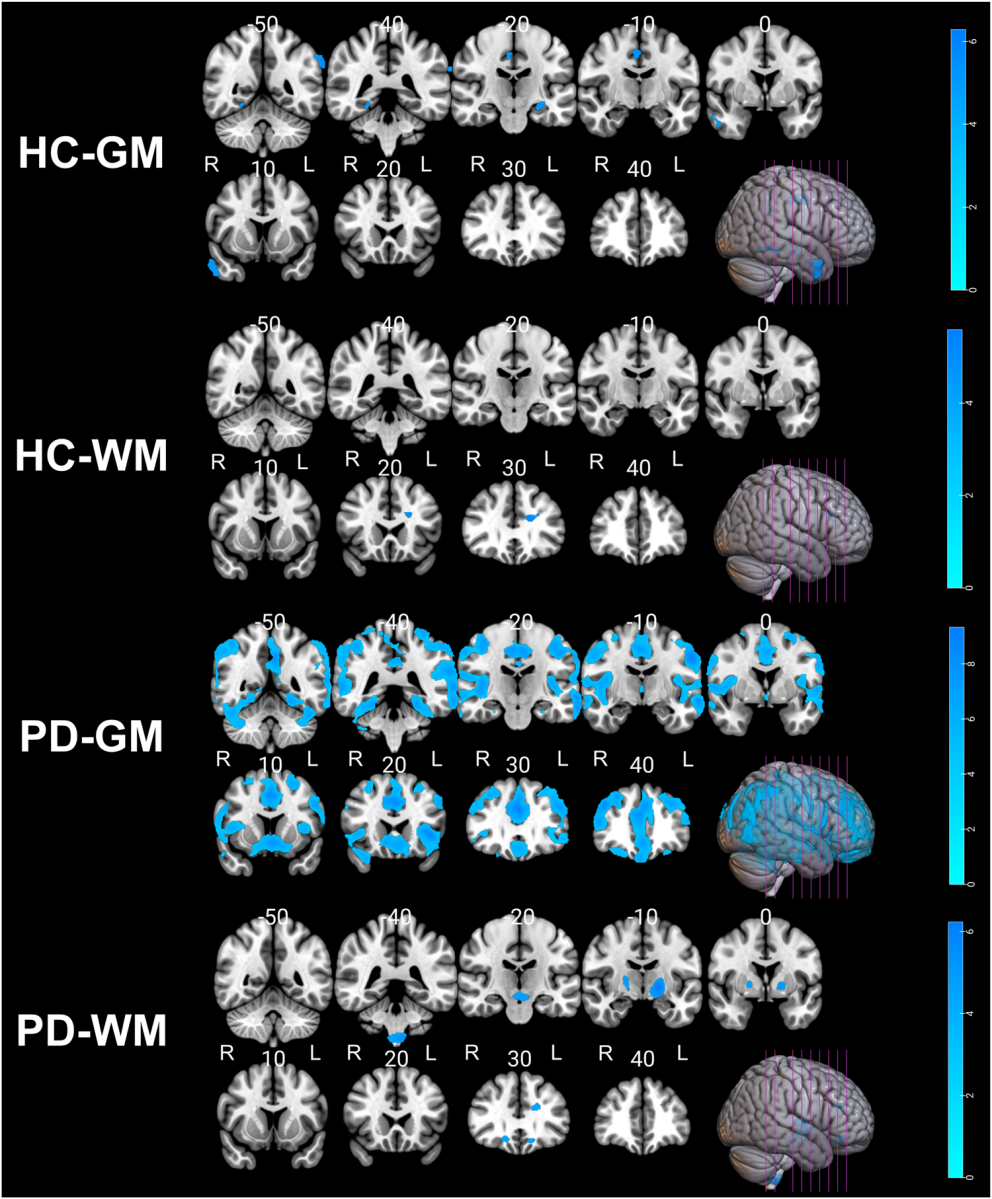
Negative association between gray and white matter volumes and Brain-PAD scores in the healthy control and PD groups. The *p* values were corrected using FWE at a threshold of *p* < 0.05, and the cluster extent threshold was set to a minimum of 100. Labels "R" and "L" stand for the right and left hemispheres, respectively.

To visualize the brain regions that significantly contributed to Brain-PAD score estimation, the Brain-PAD scores were regressed to gray matter (GM)/white matter (WM), tissue probability maps in voxel-based morphometry (VBM) analysis. As expected, negative correlations with GM/WM volumes were observed throughout the brain (Tables 8 and 9, and Fig. 3). In the healthy group, multiple regression analysis revealed a significant decrease in GM volume with increasing Brain-PAD scores in the parietal lobe, limbic lobe, middle temporal gyrus, hippocampus, parahippocampal gyrus, and frontal lobe regions. In addition, healthy subjects showed decreased WM volume within the frontal lobe and limbic lobe regions. In the PD group, we observed a significant reduction in GM volume located extensively in the primary frontal, temporal, parietal, limbic, and occipital lobes. Higher Brain-PAD scores in the PD group were associated with decreased WM volumes in the cerebellum, lentiform nucleus, medulla, midbrain, and frontal lobe. There was no positive association between Brain-PAD scores and GM/WM volumes in either the HC or PD groups.

**Table 8:** Clusters of negative gray matter and white matter alterations with brain-PAD in healthy controls (*N* = 105).

Analysis	Cluster	Region	BA	Cluster size (ml)	*P* (FWE)#	Hemisphere	MNI coordinats (*x*, *y*, *z*)	*T* value (peak voxel)
GM	1	Parietal Lobe/Supramarginal Gyrus	40	1.35	<0.001	L	−66, −46, 21	6.30
	2	Limbic Lobe/Cingulate_Mid	24/31	1.30	<0.001	R R R	3, −9, 45 6, −22, 42 8, −14, 38	6.13 5.27 4.95
	3	Middle Temporal Gyrus/Temporal_Inf	21/38	1.91	0.001	R R	51, 4, −33 57, −6, −26	6.02 4.92
	4	Hippocampus/Limbic Lobe/Parahippocampa Gyrus	–	0.79	0.002	L	−27, −15, −14	5.68
	5	Frontal Lobe/Frontal_Inf_Tri	–	0.36	0.005	L	−42, 45, 8	5.48
	6	Fusiform/Limbic Lobe/Parahippocampa Gyrus	19/37	0.70	0.005	R R	24, −52, −12 22, −44, −12	5.45 5.29
WM	1	Frontal Lobe/Limbic Lobe/Sub-Gyral	–	1.07	0.002	L	−16, 27, 24	5.63

*BA* Brodmann area, *R* right hemisphere, *L* left hemisphere, *GM* gray matter, *WM* white matter, *HC* healthy controls, *MNI* Montreal Neurological Institute, *FWE* family-wise error false discovery rate; # the *p*-value reported at peak-level based on FWE corrections.

**Table 9:** Clusters of negative gray matter and white matter alterations with Brain-PAD in Parkinson’s disease (*N* = 373).

Analysis	Cluster	Region	BA	Cluster size (ml)	P# (FWE)	Hemisphere	MNI coordinats (*x*, *y*, *z*)	*T* value (peak voxel)
GM	1	Frontal Lobe/Temporal Lobe/Parietal Lobe/Limbic Lobe	40/32/13/31/10/22/21/	338.98	<0.001	L L	−2, 27, 30 −4, −64, 15	9.39 8.87
	2	Frontal Lobe/Middle Frontal Gyrus/Superior Frontal Gyrus	10/9/46/8	19.99	<0.001	R R	40,40, 32 48,40,14	8.00 7.54
	3	Frontal Lobe/Superior Frontal Gyrus	11	0.59	<0.001	L	−28, 45, −21	5.86
	4	Frontal Lobe/Inferior Frontal Gyrus/Superior Frontal Gyrus	11	2.44	<0.001	R R	26, 36, −22 27, 46, −22	5.81 5.73
	5	Occipital Lobe/Parietal Lobe/Precuneus	19	1.42	<0.001	R R	32, −82, 38 34, −90, 24	5.79 5.62
	6	Frontal Lobe/Middle Frontal Gyrus	10	1.33	<0.001	L L	−50, 48, 0 −46, 54, −6	5.71 5.70
WM	1	Sub-lobar/Midbrain/Lentiform Nucleus	–	4.81	<0.001	L	−16, −9, −2	6.24
	2	Cerebellum/Medulla/Left Brainstem	–	1.85	<0.001	L R	−9, −40, −60 9, −40, −60	5.61 4.80
	3	Cerebrum/Sub-lobar/Lentiform Nucleus	–	1.23	0.002	R	20, −10, 6	5.20
	4	Frontal Lobe/Middle Frontal Gyrus	47	0.61	0.004	R	20, 33, −14	4.98
	5	Cerebrum/Frontal Lobe/ Anterior Cingulate/Limbic Lobe	–	0.70	0.008	L	−16, 30, 21	4.84
	6	Rectus/Medial Frontal Gyrus	47	0.37	0.014	L	−12, 28, −15	4.70

*BA* Brodmann area, *R* right hemisphere, *L* left hemisphere, *GM* gray matter, WM white matter, *PD* Parkinson’s disease, *MNI* Montreal Neurological Institute, *FWE* family-wise error false discovery rate, *#* the *p*-value reported at peak-level based on FWE corrections.

**Fig. 4:**
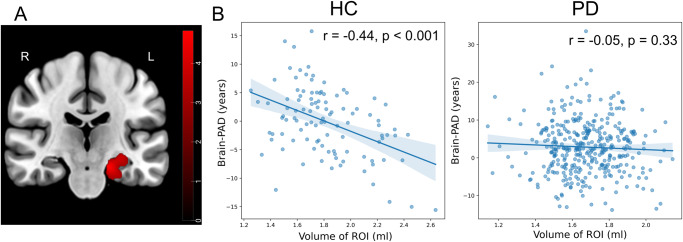
Reduced correlation between brain-PAD and regional GM volume in PD. **A** The brain region shows a significant difference in regression slopes between the HC and PD groups in GM. **B** The correlation between GM volumes in the region that was identified to be significant and Brain-PAD within each group. Labels ‘R’ and ‘L’ stand for the right and left hemispheres, respectively. The color bar corresponds to *t*-test values.

Figure 4 illustrates the regions where the regression slopes between the HC and PD groups diverged in GM, as well as the correlation between GM volumes and Brain-PAD in each group. A significant cluster, consisting of 1200 voxels, was predominantly located in the left Parahippocampal Gyrus, extending to Hippocampus and Amygdala. Notably, the HC group exhibited a significant negative correlation between GM volumes and Brain-PAD in this region (*r* = −0.44, *p* < 0.001), whereas the PD group did not demonstrate such a correlation (Fig. 4B; *r* = −0.05, *p* < 0.33). There was no significant difference in the regression slopes between the HC and PD groups in WM.

**Fig. 5:**
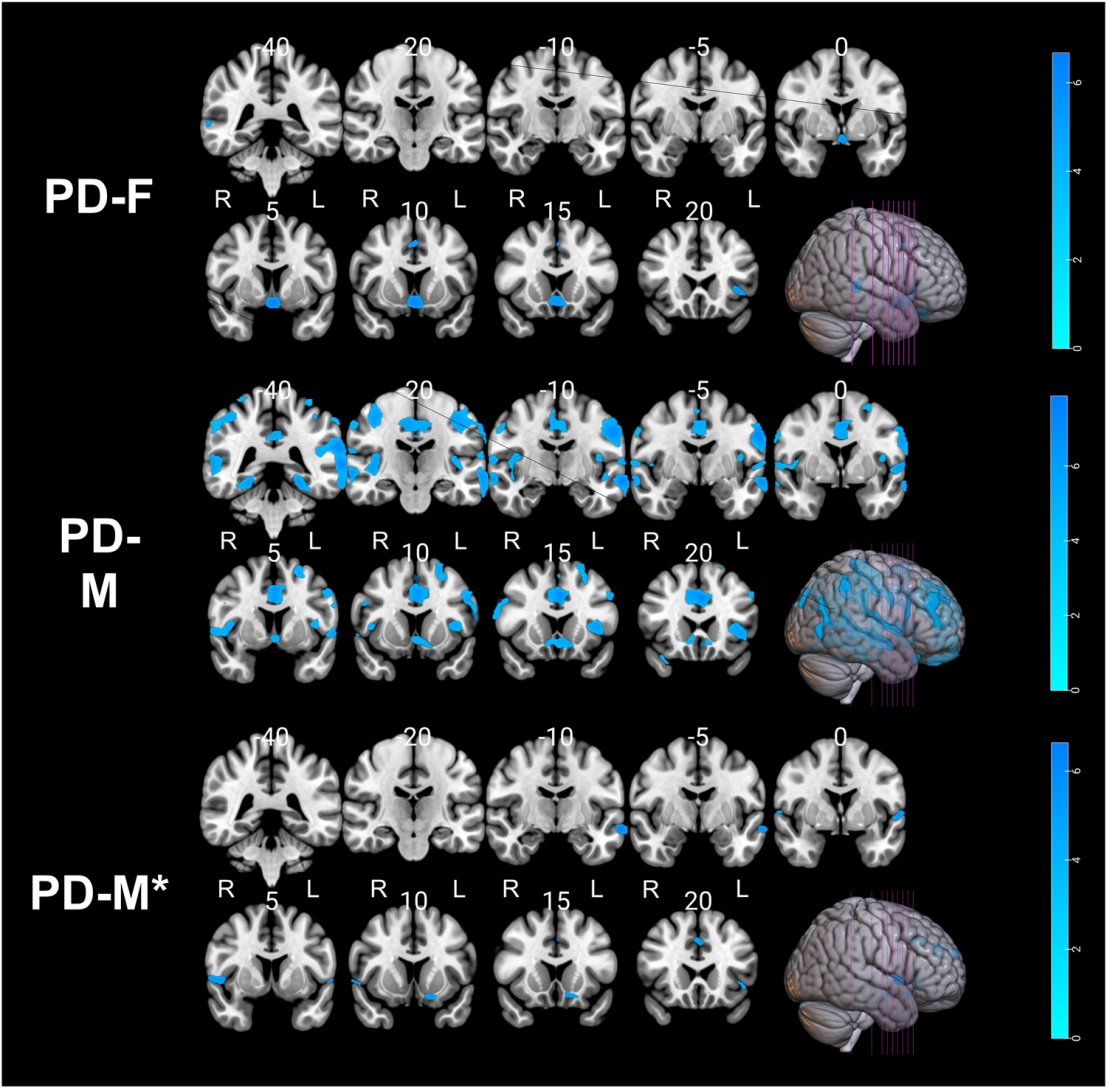
Negative association between gray matter volumes and Brain-PAD scores in the PD group within each sex. The *p* values were corrected using FWE at a threshold of *p* < 0.05, and the cluster extent threshold was set to a minimum of 100. Labels "R" and "L" stand for the right and left hemispheres, respectively.

Figure 5 illustrates the association between volumes of GM/WM and Brain-PAD scores within each sex (i.e., PD-F, PD-M, PD-M*). Notably, we observed a significant reduction in GM volume across all subgroups as Brain-PAD scores increased (Table 10 and Fig. 5). In the PD-M group, a similar topology of negative correlation between Brain-PAD scores and GM volumes was observed as identified in the regression analysis using the whole PwP (Fig. 3), while the lesser degree of association was observed in PD-M* and PD-F, potentially due to the smaller sample size. No negative correlation was observed between Brain-PAD scores and WM volumes in all PD subgroups. Similarly, no positive association was observed between Brain-PAD scores and GM/WM volumes in all PD subgroups. The difference in regression slopes between the PD-F and PD-M* groups was not statistically significant for either GM or WM modalities.

**Table 10:** Clusters of negative gray matter alterations with Brain-PAD in in PD group with respect to the sex categories (PD-F, *N* = 129; PD-M, *N* = 244; PD-M*, *N* = 129).

Analysis	Cluster	Region	BA	Cluster size (ml)	P# (FWE)	Hemisphere	MNI coordinatsv (*x*, *y*, *z*)	T value (peak voxel)
PD-F	1	Frontal Lobe/Anterior Cingulate/Limbic Lobe	25	2.66	<0.001	L	0, 4, −14	6.73
	2	Temporal Lobe/Middle Temporal Gyrus	22	0.83	<0.001	R R	62, −36, 3 48, −32,10	6.02 5.29
	3	Frontal Lobe/Inferior Frontal Gyrus/Insula	–	0.87	<0.001	L	−39, 21, −2	5.86
	4	Frontal Lobe/Inferior Frontal Gyrus	11	1.15	<0.001	L	−15, 28, −22	5.59
	5	Cingulate Gyrus/Limbic Lobe	32	0.37	<0.001	R	3, 9, 44	5.35
PD-M	1	Temporal Lobe/Parietal Lobe/Frontal Lobe/Postcentral Gyrus	40/21/22	52.49	<0.001	L L L	−63, −38, 21 −63, −12, −10 −66, −38, 3	7.93 7.88 7.44
	2	Limbic Lobe/Cingulate Gyrus/Frontal Lobe/Precuneus	31/32/24	56.80	<0.001	R R ?	26, −33, −18 3, 26, 34 0, 46, 14	7.61 7.48 7.38
	3	Parietal Lobe/Precentral Gyrus/Inferior Parietal Lobule	4/40	18.53	<0.001	R R R	57, −46, 44 42, −20, 2 39, −20, 9	6.83 6.28 6.23
	4	Fusiform/Limbic Lobe/Parahippocampa Gyrus	36/19	4.34	<0.001	L L L	−24, −39, −14 34, −34,−22 27, −63,−12	6.58 5.45 5.43
	5	Frontal Lobe/Middle Frontal Gyrus/Superior Frontal Gyrus	9/46/10	6.22	<0.001	L L L	−32, 42, 33 −44, 30, 28 −30, 51, 21	6.57 6.30 5.84
PD-M*	1	Temporal Lobe/Middle Temporal Gyrus	21	1.16	<0.001	L	−62, −10, −12	6.66
	2	Frontal Lobe/Medial Frontal Gyrus	9	0.81	<0.001	L	−4, −42, 28	6.18
	3	Cerebrum/Superior Temporal Gyrus/Temporal Lobe	–	0.47	0.001	L	−57, 0, 2	5.77
	4	Frontal Lobe/Subcallosal Gyrus/Inferior Frontal Gyrus	47	0.55	0.001	L	−14, 12, −15	5.68
	5	Cerebrum/Frontal Lobe/Superior Temporal Gyrus	22	0.87	<0.001	R R	48, 4, 0 57,4,2	5.67 5.33

*BA* Brodmann area, *R* right hemisphere, *L* left hemisphere, *GM* gray matter, *WM* white matter, *PD* Parkinson’s disease, *MNI* Montreal Neurological Institute, *FWE* family-wise error false discovery rate, *#* the *p*-value reported at peak-level based on FWE corrections.

## Discussion

In this study, we developed a brain age estimation model based on multi-site and multi-scanner datasets, such as IXI, OASIS, and PPMI, demonstrating the generalizability of our results. Of note, the magnetic resonance imaging (MRI) processing technique used in this study has been thoroughly examined and found to be suitable for multi-center and multi-scanner studies^19^. The model accuracy measured by MAE (4.63–4.72 years) was well comparable with those of other studies^14,20^. We then applied this methodology to investigate the sex-specific association between accelerated brain aging and PD pathology. We found that PwP had a significantly greater Brain-PAD than healthy controls (*t*(476) = 3.94, *p* <<0.0011), which is in line with what has been observed in previous studies^17,18^. Of note, the elevated Brain-PAD does not represent specific PD pathology, but it may be associated with a more general measure of cognitive impairment that is differently affected in each individual’s distinctive disease processes. The increased inter-individual variability (decreased R2) of Brain-PAD in PD groups confirms the heterogeneity of this relationship.

Brain-PAD was significantly greater in male PD compared to females (*t*(371) = 2.26, *p* = 0.024). This sex difference was still significant when propensity score-matched males were used (*t*(256) = 2.50, *p* = 0.012). Of note, our brain age prediction model did not show a significant difference in Brain-PAD between male and female HC (training set: *t*(947) = 0.52, *p* = 0.60; hold-out set: *t*(103) = 0.56, *p* = 0.57). This result would suggest that the male sex is more influenced by disease-accelerated brain atrophy associated with aging in PD^7,21^.

Several previous studies have investigated sex differences in brain structure in PD using structural brain imaging, with some variability in the results^7^. In established PD, increased atrophy has been observed in males in extensive cortical and subcortical regions, as measured using VBM as well as cortical thickness^7,21^. According to a study conducted on the PPMI dataset, male de novo PwP exhibit greater cortical thinning in the pre- and post-central gyrus and lower volume of the basal ganglia, thalamus, and brainstem compared to their female counterparts^22^. Male sex has also been shown to be a significant predictor of greater atrophy and clinical severity in PD in the PPMI dataset^23^. While we did not directly compare brain structure between male and female PwP in this study, we found that Brain-PAD in the PD-M* was significantly higher than in the PD-F group (Table 2). The clinical relevance of Brain-PAD in PD, in general, has been demonstrated by its significant correlation with the UPDRS-III score (Table 3), supporting the idea that brain age measurements can provide a clinically relevant metric for ascertaining the severity and extent of PD-induced neurodegeneration. As expected, UPDRS-III correlated with the patient’s age and disease duration in this study cohort. While this correlation persisted when looking at just the PD-M group, the statistical significance was no longer observed when analyzed within PD-F or PD-M*. It is unknown if the non-significance in PD-M* is due to the low sample size (*n* = 244 vs. 129) or unaccounted bias that can be introduced in the propensity score matching process^24^.

Our analysis showed key sex differences in the relationship between accelerated brain age and cognitive symptoms of PD. Interestingly, a lower MoCA score was significantly associated with Brain-PAD only in male PD (both PD-M and PD-M*, *p* < 0.04), while it was not significant in female PwP (*p* = 0.782). Additionally, visuospatial acuity (BJLO score) was negatively correlated with Brain-PAD only in the pooled PD group and the PD-M* group, indicating this correlation is mainly driven by male PwP. Male sex is significantly associated with a higher incidence of MCI in de novo PD, as well as more rapid progression to dementia longitudinally^5,6,25^. More specifically, male PwP exhibits impairments in overall cognition, processing speed, and working memory than females, whereas females tend to display more severe deficits in visuospatial function^6,26^, although relatively better visuospatial function in males is also observed in normal aging^27^. Deficit in visuospatial function in males, according to our data, is potentially tied to an accelerated brain aging phenotype, which is expressed to a greater degree in males than in females and may partly underlie the observed sex differences in cognitive symptoms.

When looking at the association between Brain-PAD and other symptoms of PD, our regression analysis revealed significant sex differences, particularly in non-motor symptoms of PD, such as cognition and REM sleep behavior disorder scores (RBD) (Table 4). The RBD score was significantly correlated with the Brain-PAD score in our pooled cohort of PwP. Additionally, when we looked at females with PD and propensity-matched males with PD separately, we found that the correlation between RBD and Brain-PAD was significant only in the male group. RBD sleep disorder is one of the most common prodromal symptoms of PD and is a strong risk factor for the development of PD or other synucleinopathies^28^. Furthermore, the co-occurrence of RBD sleep disorder in individuals with PD is linked with inferior cognitive functioning, heightened levels of depression, and apathetic symptoms^28,29^. The presence of RBD in PD is often accompanied by a negative prognosis due to the correlation with autonomic dysfunction, heightened disease burden, and increased mortality rates, as evidenced by various studies^30^.

Very little is understood about the neuropathology of RBD, but it appears that the key neuronal networks in the brainstem that regulate skeletal muscle atonia during sleep are selectively vulnerable to synucleopathy^31^. While some studies have noted an increased incidence in male PwP^32^, others have found no apparent sex difference in the presentation of RBD^30^. Further investigation has shown a difference in the presentation of REM-sleep behavior disorder, with male PwP presenting with a significantly more violent form with more vigorous motor activity, which could possibly lead to a detection bias for RBD in males^33^. A recent study found that male PwP with RBD have significantly worse subcortical brain atrophy compared to female PwP, and these sex differences are greater than those observed when comparing male and female PwP without RBD^34^. Moreover, male PwP with PD-RBD have severe cognitive symptoms. This, along with the results of our study, indicates that RBD coincides with greater macroscopic structural alterations in males compared to females PwP.

To visualize the brain regions that mainly contributed to the Brain-PAD score estimation, a regression model was constructed to examine the correlation between Brain-PAD scores and volumes of GM and WM. Within the HC group (i.e., hold-out set), an elevation in Brain-PAD demonstrated a significant association with decreased GM volume, particularly in the limbic area. Furthermore, an observed reduction in WM within the limbic region corresponded to increasing Brain-PAD in the HC group, suggesting a potential link between elevated Brain-PAD and cognitive decline as well as deficits in emotional processing among older HC individuals.

In the PD group, there was a notably greater magnitude of GM changes in response to increasing Brain-PAD compared to WM. These findings suggest a stronger association between escalated Brain-PAD and the degeneration of neuronal cell bodies, dendrites, and glial cells rather than myelinated nerve fibers (axons) among individuals with PD. Regarding the specific brain regions associated with increasing Brain-PAD in PD, notable GM changes were primarily localized within the Frontal Lobe, Limbic Lobe, Superior Frontal Gyrus, and Brodmann Areas 31 and 10/46 (Dorsolateral Prefrontal Cortex). These regions are known to be involved in cognitive and executive functions. Additionally, the study demonstrated significant WM changes in response to greater Brain-PAD in PD, primarily concentrated in the midbrain, basal ganglia, and cerebellum regions. These areas are particularly relevant to motor control, coordination, balance, and posture, as well as the timing and coordination of movements (Fig. 3).

In addition, our investigation included conducting regression analyses involving the interaction between GM/WM volume and Brain-PAD scores, with the aim of identifying dissimilarities between the HC and PD groups. We observed a significant distinction in the regression slopes between the HC and PD groups in relation to GM, primarily localized in the parahippocampal/hippocampal/amygdala area (Fig. 4A). As expected, we found a significant correlation between GM volume in this ROI and Brain-PAD values within the HC group suggesting a significant contribution of this region’s atrophy toward the overall brain age estimation (Fig. 4B). However, this significant correlation was eliminated within the PD group, potentially suggesting that the disease-related degeneration interfered with the normal aging-related degeneration in this limbic area.

The cause of sex differences in PD symptomatology and incidence between male and female PwP is not well understood but may involve a neuroprotective effect of estrogens modulating neuroinflammation, metabolism, and signaling of the nigrostriatal dopamine system, along with other neurodevelopmental factors^35^. This is supported by the observation that the age of menopause, duration of fertile life, and levels of female sex hormones significantly affect the incidence and risk of PD in females^36,37^. Estrogens may also prevent aggregation and defibrillation of alpha-synuclein, reducing the deposition of Lewy bodies^38–40^. This mechanism could help explain the differences in regional atrophy and white matter integrity throughout the brain between sexes and would lead to the enhanced pattern of regional cortical atrophy among male PwP, as reported in other studies^7^. These points could potentially explain why males with PD had faster brain aging than females in our study. It is worth mentioning that, despite the fact that male sex is associated with poorer prognosis, cognitive symptoms, and higher prevalence of PD, the converse holds true in the case of AD^41^. In the context of AD, women tend to perform worse on neurocognitive tests and exhibit a higher degree of atrophy, as compared to men^41,42^. This observation indicates that the higher incidence of disease and relatively worse outcomes for males in cognitive and motor symptoms in PD are not only due to underlying sex differences in healthy aging or a generalized increased vulnerability to neurodegeneration in males but also an increased vulnerability specifically to synucleinopathies. Further, longitudinal studies are necessary to accurately quantify the differences in brain aging between males and females with PD.

Our research furthers our insight into the distinctive neurological characteristics seen in male PwP. Our study has revealed a remarkable neurological presentation that appears as an accelerated aging-related phenotype in brain structure. By examining the brain structure of male PwP, we noticed distinct patterns that suggest an accelerated aging process. Changes in the brain’s structure appear to indicate a disruption to the typical aging process in men with PD. Grasping the diverse neurological features in PwP of different sexes is imperative for devising targeted interventions and treatments that meet their particular needs. By recognizing the accelerated aging-related changes in the brain structure, we can potentially discover novel therapeutic targets (such as neuroprotective strategies, inflammation and oxidative stress, dopaminergic system modulation, and lifestyle and environmental interventions) to decrease the impact of PD on brain health and its progression in this population.

A limitation of our study is that we did not directly examine the interaction effects between sexes and Brain-PAD. The investigated models were already too complex with ≥5 predictor variables, and these predictor variables were correlated with each other. Therefore, adding interaction terms can exacerbate multicollinearity and result in unstable models. The limited sample size was also a factor, and thus, we decided not to include the interaction terms of sex × Brain-PAD. Indeed, in our unreported observations, including this interaction term did not improve the model fitting in any of the regression analyses. Another limitation is that there was a significant overlap of Brain-PAD at the individual level, and thus, our present results should only be interpreted at the group level. It may be premature to use Brain-PAD as a subject-specific progression marker in clinical practice. An expanded dataset of the PPMI with longitudinal follow-up scans may allow us to address these issues in future studies.

## Methods

### Participants and MRI acquisition

To train and validate a model that estimates Brain Age, we employed a total of 1054 T1-weighted (T1w) MRI scans from HCs obtained from the Open Access Series of Imaging Studies (OASIS) (https://www.oasis-brains.org/), the IXI (http://brain-development.org/ixi-dataset/), and the PPMI databases. Each database was approved by an ethics committee for human experimentation before the study commenced, and the participants were provided written information. All healthy controls were free from cognitive impairment or any neurological diseases, as per the databases. Their mean age was 49.15 ± 19.06, of which 53% were female. A brain age estimation model was trained using these HCs (see Section “Brain age across different groups” for details).

To test whether brain age metric was differently correlated with clinical symptoms severities between sexes, a total of 373 individuals diagnosed with PD were included in the study, with a mean age of 61.37 ± 9.81 years and an age range of 33–85 years. Among the PD participants, 34% were female. Baseline T1w MRI scans and clinical data were obtained from the PPMI database in September 2022, and brain age was calculated for each subject.

In addition to demographic characteristics (i.e., age, education, age at diagnosis, and disease duration), we collected clinical measures, including motor symptom scores (i.e., UPDRS-III (total), UPDRS-III (total rigidity), and UPDRS-III (total tremor)), non-motor symptom scores (i.e., MoCA, ESS, Letter Number Sequencing, REM, BJLO, HVLT delayed recall, HVLT delayed recognition, olfactory testing, Symbol Digit Modalities Score), mood (i.e., anxiety and GDS), CSF biomarkers (i.e., alpha-synuclein (a-syn), amyloid-beta, and CSF p-tau (2016 assay)) for PwP. We also included measurements of the left and right Striatal Binding Ratios (SBRs) derived from SPECT for the caudate and putamen regions. SBR values were obtained from the PPMI database. Briefly, SPECT data underwent HOSEM reconstruction in the HERMES system without filtering. Reconstructed files were processed in PMOD. Images underwent attenuation correction and a Gaussian 3D filter. They were normalized to the Montreal Neurologic Institute (MNI) space. Regions of interest (ROIs) were defined for the caudate, putamen, and occipital cortex (reference tissue). SBR was calculated by subtracting one from the ratio of the target to reference regions. Detailed information regarding SBR contribution can be found at https://www.ppmi-info.org.

In the group under study, the number of individuals diagnosed with PD consisted of 244 males and 129 females. To ensure that the severity of the disease was consistent across both sexes in the PD group, we identified a set of male PwP (PD-M*, *N* = 129) through propensity score matching from the larger group of 244 male PwP. These PwP were matched based on actual age, education level, age of diagnosis, UPDRS-III (total), UPDRS-III (rigidity), UPDRS-III (tremor), MoCA, REM, and ESS scores. Propensity score matching was conducted using the *pymatch* package in Python (https://github.com/benmiroglio/pymatch), which employs logistic regression models to generate propensity scores and match two groups.

### Image processing and brain age estimation model

Brain age estimation was performed based on T1-weighted MRI data. The VBM technique implemented in CAT12 toolbox (http://www.neuro.uni-jena.de/cat/), as an extension of the Statistical Parametric Mapping (SPM12) software package (https://www.fil.ion.ucl.ac.uk/spm/software/spm12/), was used for preprocessing T1-weighted MRI scans with the default set of parameters, including DARTEL normalization and modulation for nonlinear components, as described in^43^. Based on the VBM technique, we generated the density images of GM, WM, and CSF for each T1w MRI scan. We then smoothed these images with a 4-mm kernel. The smoothed GM, WM, and CSF images were re-sampled to an 8-mm isotropic spatial resolution, resulting in 3747 voxels per volume. We also computed the total volumes of GM, WM, and CSF for each subject using the CAT12 toolbox, as well as the total intracranial volume (TIV). This procedure was applied to both the training and testing datasets. Visual assessment of the quality of MRI processing and segmentation was performed for all MRI scans, followed by a quality assurance check using the “Check homogeneity function” in CAT12.

To estimate the values of brain age, we utilized a support vector regression (SVR) algorithm with a linear kernel that was implemented in MATLAB_R2020b (The Mathworks, Natick, MA, USA). To train the model, we randomly selected 90% of healthy controls (HCs), consisting of 949 individuals with a mean age of 49.75 ± 18.96 years and an age range of 18–94 years, of which 54% were female. The remaining 10% of HCs, totaling 105 individuals with a mean age of 48.62 ± 19.14 years and an age range of 18–93 years, of which 53% were female, were held out as a sample for validation purposes. In the prediction model, the independent variables comprised the voxel intensities of GM, WM, and CSF, along with the variables of sex, scanner vendor, field strength, TIV, and total brain volumes of GM, WM, and CSF (Table 11). The dependent variable was the actual age. The MAE, root mean square error (RMSE), and coefficient of determination (*R*^2^) between actual age and model-estimated age were used to assess prediction performance in the training set (*N* = 949) using a 10-fold cross-validation strategy, as well as in the hold-out control set (*N* = 105). The Brain-PAD (i.e., model-estimated age minus actual age) was also calculated in the form of the mean and a 95% confidence interval (CI). The bias-free brain age values were computed using a validated bias adjustment scheme, which is detailed in^44^. The bias adjustment approach utilized in this study is publicly available at: https://github.com/medicslab/Bias_Correction. The intention of bias adjustment is to minimize the age-dependency of the predicted results, which could be due to regression dilution bias^44^. Next, the final prediction model was developed using the entire training set (*N* = 949) and applied to hold-out sets (HC: *N* = 105; PD: *N* = 373) to compute the Brain-PAD for those sets. A Brain-PAD value close to zero (i.e., estimated age ≅ actual age) stands for the point that the subject being studied follows a healthy brain aging trajectory. A negative Brain-PAD value (i.e., estimated age < actual age) indicates a younger-looking brain, and a positive Brain-PAD value (i.e., estimated age > actual age) indicates an older-looking brain.

**Table 11:** List of independent variables used in a brain age prediction model.

Variable	Description	Number of attributes
Voxel intensities of GM	Intensity values of gray matter voxels	3747
Voxel intensities of WM	Intensity values of white matter voxels	3747
Voxel intensities of CSF	Intensity values of cerebrospinal fluid voxels	3747
Total brain volume of GM	Total volume of gray matter in the brain	1
Total brain volume of WM	Total volume of white matter in the brain	1
Total brain volume of CSF	Total volume of cerebrospinal fluid in the brain	1
Total intracranial volume	Total volume of the brain	1
Sex	Biological sex of the participant	1
Scanner vendor	Manufacturer or brand of the scanning equipment	1
Field strength	Magnetic field strength of the scanner	1

### Statistical analysis

The mean Brain-PAD between the hold-out sets was examined using an independent Student’s *t*-test. We used multiple linear regression models to examine whether Brain-PAD is able to predict the clinical variables in PD. The models that have been tested are:

1. Motor symptom severity (UPDRS-III) ~ 1 + Brain-PAD + age + disease duration + education + sex
2. Non-motor symptom severity (see Table 1) ~ 1 + Brain-PAD + age + disease duration + education + sex + UPDRS-III

UPDRS-III was considered in equation (2) to examine whether it influences the severity of non-motor symptoms. For mood symptoms, the severity of cognitive symptoms assessed by the MoCA was also modeled:

(3) Mood symptom severity (anxiety (i.e., the state-trait anxiety inventory test) and GDS) ~ 1 + Brain-PAD + age + disease duration + education + sex + UPDRS-III + MoCA

We repeated the above regression models for males and females separately to assess the differential association of Brain-PAD and clinical symptom severity in each sex. Likewise, UPDRS-III and MoCA were considered in Eq. (3) to examine whether motor and non-motor symptoms influence mood status in PD. All multiple linear regression models were analyzed in MATLAB using the *regstats* function. For each model, we reported the adjusted *R*^2^, *F*-statistic, and *p*-value. The false discovery rate (FDR) strategy was employed to adjust the *p*-values. For all statistical examinations, a *p*-value of less than 0.05 was considered significant. The predictor coefficients and their *t*- and *p*-values were reported if the overall prediction model was significant. The statistics of non-significant models are also reported for the completeness of the tables, but these results are not discussed in detail. For each statistical analysis, we excluded subjects with missing data.

To visualize the brain regions that were specifically associated with advanced brain age, we used multiple regression analysis in SPM12 to predict GM/WM volumes with Brain-PAD scores as an independent variable in each group (i.e., HC and PD), separately. To identify the brain regions that were differently correlated with Brain-PAD scores in PD vs. HC, regression coefficients were contrasted between the groups. For these analyses, the peak-level *p*-values were considered significant at 0.05 after being corrected with a family-wise error. We only considered clusters with >100 voxels. All regression analyses incorporated age, sex, and TIV as covariates.

### Supplementary information

Related Manuscript File

## Data Availability

We implemented the brain age estimation and bias adjustment approach based on our previously validated code, which can be accessed from the public repository at https://github.com/medicslab/Bias_Correction.

## Data availability

Data used in the preparation of this article were obtained from three open-access datasets: the Open Access Series of Imaging Studies (OASIS; http://www.oasis-brains.org), the IXI (http://www.brain-development.org/), and the Parkinson’s Progression Markers Initiative (PPMI; www.ppmi-info.org/data). The data used in this study were downloaded on September 10, 2022.

## References

[1] Mu J (2017). Parkinson’s disease subtypes identified from cluster analysis of motor and non-motor symptoms. Front. Aging Neurosci..

[2] Han JW (2018). Psychiatric manifestation in patients with Parkinson’s disease. J. Korean Med. Sci..

[3] Georgiev D, Hamberg K, Hariz M, Forsgren L, Hariz GM (2017). Gender differences in Parkinson’s disease: a clinical perspective. Acta Neurol. Scand..

[4] Elbaz A (2002). Risk tables for Parkinsonism and Parkinson’s disease. J. Clin. Epidemiol..

[5] Cholerton B (2018). Sex differences in progression to mild cognitive impairment and dementia in Parkinson’s disease. Parkinsonism Relat. Disord..

[6] Liu R (2015). Potential sex differences in nonmotor symptoms in early drug-naive Parkinson disease. Neurology.

[7] Tremblay C (2020). Sex effects on brain structure in de novo Parkinson’s disease: a multimodal neuroimaging study. Brain.

[8] Yadav SK (2016). Gender-based analysis of cortical thickness and structural connectivity in Parkinson’s disease. J. Neurol..

[9] Crivello F, Tzourio-Mazoyer N, Tzourio C, Mazoyer B (2014). Longitudinal assessment of global and regional rate of grey matter atrophy in 1,172 healthy older adults: modulation by sex and age. PloS ONE.

[10] Rodriguez M, Rodriguez‐Sabate C, Morales I, Sanchez A, Sabate M (2015). Parkinson’s disease as a result of aging. Aging Cell.

[11] Moeller J, Eidelberg D (1997). Divergent expression of regional metabolic topographies in Parkinson’s disease and normal ageing. Brain.

[12] Mishra S, Beheshti I, Khanna P (2021). A review of neuroimaging-driven brain age estimation for identification of brain disorders and health conditions. IEEE Rev. Biomed. Eng..

[13] Beheshti I, Nugent S, Potvin O, Duchesne S (2021). Disappearing metabolic youthfulness in the cognitively impaired female brain. Neurobiol. Aging.

[14] Sone D (2021). Neuroimaging-based brain-age prediction in diverse forms of epilepsy: a signature of psychosis and beyond. Mol. Psychiatry.

[15] Beheshti I (2023). Cocaine destroys gray matter brain cells and accelerates brain aging. Biology.

[16] Koutsouleris N (2014). Accelerated brain aging in schizophrenia and beyond: a neuroanatomical marker of psychiatric disorders. Schizophr. Bull..

[17] Eickhoff CR (2021). Advanced brain ageing in Parkinson’s disease is related to disease duration and individual impairment. Brain Commun..

[18] Beheshti I, Mishra S, Sone D, Khanna P, Matsuda H (2020). T1-weighted MRI-driven brain age estimation in Alzheimer’s disease and Parkinson’s disease. Aging Dis..

[19] Dadar M, Duchesne S (2020). Reliability assessment of tissue classification algorithms for multi-center and multi-scanner data. Neuroimage.

[20] Cole JH (2018). Brain age predicts mortality. Mol. Psychiatry.

[21] Alqarni A (2017). Structural brain sex differences in Parkinson’s disease: a voxel-based morphometry study. J. Neurol. Disord..

[22] Oltra J (2022). Sex differences in brain and cognition in de novo Parkinson’s disease. Front. Aging Neurosci..

[23] Zeighami Y (2019). A clinical-anatomical signature of Parkinson’s disease identified with partial least squares and magnetic resonance imaging. Neuroimage.

[24] King G, Nielsen R (2019). Why propensity scores should not be used for matching. Polit. Anal..

[25] Aarsland D (2017). Cognitive decline in Parkinson disease. Nat. Rev. Neurol..

[26] Bakeberg MC (2021). Differential effects of sex on longitudinal patterns of cognitive decline in Parkinson’s disease. J. Neurol..

[27] McCarrey AC, An Y, Kitner-Triolo MH, Ferrucci L, Resnick SM (2016). Sex differences in cognitive trajectories in clinically normal older adults. Psychol. Aging.

[28] Postuma RB, Lang A, Gagnon J, Pelletier A, Montplaisir J (2012). How does parkinsonism start? Prodromal parkinsonism motor changes in idiopathic REM sleep behaviour disorder. Brain.

[29] Mahmood Z (2020). REM sleep behavior disorder in Parkinson’s disease: effects on cognitive, psychiatric, and functional outcomes. J. Int. Neuropsychol. Soc..

[30] Kim Y (2018). REM sleep behavior disorder portends poor prognosis in Parkinson’s disease: a systematic review. J. Clin. Neurosci..

[31] Boeve BF (2007). Pathophysiology of REM sleep behaviour disorder and relevance to neurodegenerative disease. Brain.

[32] Zhu R-l, Xie C-j, Hu P-p, Wang K (2017). Clinical variations in Parkinson’s disease patients with or without REM sleep behaviour disorder: a meta-analysis. Sci. Rep..

[33] Bjørnarå KA, Dietrichs E, Toft M (2013). REM sleep behavior disorder in Parkinson’s disease–is there a gender difference?. Parkinsonism Relat. Disord..

[34] Oltra J (2022). Sex differences in brain atrophy and cognitive impairment in Parkinson’s disease patients with and without probable rapid eye movement sleep behavior disorder. J. Neurol..

[35] Gillies GE, Pienaar IS, Vohra S, Qamhawi Z (2014). Sex differences in Parkinson’s disease. Front. Neuroendocrinol..

[36] Nitkowska M, Czyżyk M, Friedman A (2014). Reproductive life characteristics in females affected with Parkinson’s disease and in healthy control subjects–a comparative study on Polish population. Neurolog. Neurochir. Pol..

[37] Miller IN, Cronin‐Golomb A (2010). Gender differences in Parkinson’s disease: clinical characteristics and cognition. Mov. Disord..

[38] Hirohata M, Ono K, Morinaga A, Ikeda T, Yamada M (2009). Anti-aggregation and fibril-destabilizing effects of sex hormones on α-synuclein fibrils in vitro. Exp. Neurol..

[39] Rajsombath MM, Nam AY, Ericsson M, Nuber S (2019). Female sex and brain-selective estrogen benefit α-synuclein tetramerization and the PD-like motor syndrome in 3K transgenic mice. J. Neurosci..

[40] Fox S (2022). Estrogen-related receptor gamma regulates mitochondrial and synaptic genes and modulates vulnerability to synucleinopathy. npj Parkinson’s Dis..

[41] Ferretti MT (2018). Sex differences in Alzheimer disease—the gateway to precision medicine. Nat. Rev. Neurol..

[42] Benke T (2013). Cognition, gender, and functional abilities in Alzheimer’s disease: how are they related?. J. Alzheimer’s Dis..

[43] Farokhian F, Beheshti I, Sone D, Matsuda H (2017). Comparing CAT12 and VBM8 for detecting brain morphological abnormalities in temporal lobe epilepsy. Front. Neurol..

[44] Beheshti I, Nugent S, Potvin O, Duchesne S (2019). Bias-adjustment in neuroimaging-based brain age frameworks: a robust scheme. Neuroimage Clin..

