# Supplementary material for: Differences in brain aging between sexes in Parkinson’s disease
Source: NPJ Parkinsons Dis. 2024 Feb 14;10:35. doi: 10.1038/s41531-024-00646-w (PMC10866976; doi:10.1038/s41531-024-00646-w)
Supplement: Supplementary file 1 — Related Manuscript File [file 41531_2024_646_MOESM1_ESM.pdf]

nature portfolio

Corresponding author(s): Ji Hyun Ko & Iman Beheshti

Last updated by author(s): Dec 13, 2023

Reporting Summary

Nature Portfolio wishes to improve the reproducibility of the work that we publish. This form provides structure for consistency and transparency in reporting. For further information on Nature Portfolio policies, see our Editorial Policies and the Editorial Policy Checklist.

Statistics

For all statistical analyses, confirm that the following items are present in the figure legend, table legend, main text, or Methods section.

n/a	Confirmed	
☐	☒	The exact sample size (n) for each experimental group/condition, given as a discrete number and unit of measurement
☒	☐	A statement on whether measurements were taken from distinct samples or whether the same sample was measured repeatedly
☒	☐	The statistical test(s) used AND whether they are one- or two-sided<br>*Only common tests should be described solely by name; describe more complex techniques in the Methods section.*
☐	☒	A description of all covariates tested
☐	☒	A description of any assumptions or corrections, such as tests of normality and adjustment for multiple comparisons
☐	☒	A full description of the statistical parameters including central tendency (e.g. means) or other basic estimates (e.g. regression coefficient) AND variation (e.g. standard deviation) or associated estimates of uncertainty (e.g. confidence intervals)
☐	☒	For null hypothesis testing, the test statistic (e.g. F, t, r) with confidence intervals, effect sizes, degrees of freedom and P value noted<br>*Give P values as exact values whenever suitable.*
☒	☐	For Bayesian analysis, information on the choice of priors and Markov chain Monte Carlo settings
☒	☐	For hierarchical and complex designs, identification of the appropriate level for tests and full reporting of outcomes
☐	☒	Estimates of effect sizes (e.g. Cohen's d, Pearson's r), indicating how they were calculated

Our web collection on statistics for biologists contains articles on many of the points above.

Software and code

Policy information about availability of computer code

Data collection	No software for data collection was used.
Data analysis	All simulations and statistical analyses were conducted in MATLAB R2020b (The MathWorks, Natick, MA, USA).

For manuscripts utilizing custom algorithms or software that are central to the research but not yet described in published literature, software must be made available to editors and reviewers. We strongly encourage code deposition in a community repository (e.g. GitHub). See the Nature Portfolio guidelines for submitting code & software for further information.

Data

Policy information about availability of data

All manuscripts must include a data availability statement. This statement should provide the following information, where applicable:

- Accession codes, unique identifiers, or web links for publicly available datasets
- A description of any restrictions on data availability
- For clinical datasets or third party data, please ensure that the statement adheres to our policy

Data used in the preparation of this article were obtained from three open-access datasets: the Open Access Series of Imaging Studies (OASIS; http://www.oasis-brains.org), the IXI (http://www.brain-development.org/), and the Parkinson’s Progression Markers Initiative (PPMI; www.ppmi-info.org/data). The data used in this study were downloaded on September 10, 2022. Each database was approved by an ethics committee for human experimentation before study commenced, and the participants provided written informed.

consent.

Research involving human participants, their data, or biological material

Policy information about studies with human participants or human data. See also policy information about sex, gender (identity/presentation), and sexual orientation and race, ethnicity and racism.

Reporting on sex and gender	For assessing a possible sex influence of brain aging, Sex-stratified analyses were performed in both cohorts. All data and observations are shown in the results section.
Reporting on race, ethnicity, or other socially relevant groupings	N/A
Population characteristics	Their mean age for healthy controls (N = 1,054) was 49.15_± 19.06, of which 53% were female.<br>A total of 373 individuals diagnosed with PD were included in the study, with a mean age of 61.37±9.81 years and an age range of 33 to 85 years. Among the PD participants, 34% were female.
Recruitment	Data used in the preparation of this article were obtained from three open-access datasets: the Open Access Series of Imaging Studies (OASIS; http://www.oasis-brains.org), the IXI (http://www.brain-development.org/), and the Parkinson's Progression Markers Initiative (PPMI; www.ppmi-info.org/data).
Ethics oversight	Each database was approved by an ethics committee for human experimentation before study commenced, and the participants provided written informed consent.

Note that full information on the approval of the study protocol must also be provided in the manuscript.

Field-specific reporting

Please select the one below that is the best fit for your research. If you are not sure, read the appropriate sections before making your selection.

☒ Life sciences ☐ Behavioural & social sciences ☐ Ecological, evolutionary & environmental sciences

For a reference copy of the document with all sections, see nature.com/documents/nr-reporting-summary-flat.pdf

Life sciences study design

All studies must disclose on these points even when the disclosure is negative.

Sample size	All patients diagnosed with Parkinson's disease from the PPMI dataset, having available T1W-MRI scans and respective clinical data, were included in this study. Regarding healthy subjects, we included individuals with available T1W-MRI scans from the OASIS, IXI, and PPMI datasets.
Data exclusions	Visual assessment was conducted to evaluate the quality of MRI processing and segmentation for all scans, leading to the exclusion of samples with low-quality MRI data. Subjects with missing data were excluded from each statistical analysis.
Replication	A validation cohort was used for validating the results of our prediction model. In this study, we developed a brain age estimation model based on multi-site and multi-scanner datasets, such as IXI, OASIS, and PPMI, demonstrating the generalizability of our results. Of note, the MRI processing technique used in this study has been thoroughly examined and found to be suitable for multi-center and multi-scanner studies.
Randomization	The PD patients were stratified into two groups based on their sex, comprising 244 males and 129 females. To ensure the comparability of disease severity between sexes in the PD group, we identified a subset of male PD patients (PD-M*, N = 129) through propensity score matching from the larger pool of 244 male patients.
Blinding	Investigators remained blinded throughout the retrospective data collection, including demographic and clinical data from both healthy controls and patients with Parkinson's disease, as well as during the subsequent analysis.

Reporting for specific materials, systems and methods

We require information from authors about some types of materials, experimental systems and methods used in many studies. Here, indicate whether each material, system or method listed is relevant to your study. If you are not sure if a list item applies to your research, read the appropriate section before selecting a response.

Materials & experimental systems

n/a	Involved in the study
☒	☐ Antibodies
☒	☐ Eukaryotic cell lines
☒	☐ Palaeontology and archaeology
☒	☐ Animals and other organisms
☐	☒ Clinical data
☒	☐ Dual use research of concern
☒	☐ Plants

Methods

n/a	Involved in the study
☒	☐ ChIP-seq
☒	☐ Flow cytometry
☐	☒ MRI-based neuroimaging

Clinical data

Policy information about clinical studies

All manuscripts should comply with the ICMJE guidelines for publication of clinical research and a completed CONSORT checklist must be included with all submissions.

Clinical trial registration	Clinical data utilized in this study was sourced from the PPMI dataset. PPMI is an ongoing international, multicenter, observational study initiated in June 2010, with approval from the institutional review board at each participating site, and participants providing written informed consent.
Study protocol	Study protocol and manuals are available at www.ppmi-info.org/ study-design.
Data collection	We downloaded samples with available baseline MRI scans and clinical data. The data utilized in this study were retrieved on September 10, 2022
Outcomes	For each subject under study, we measured Brain-PAD, a metric used to assess global brain health. Subsequently, for each group of patients, we conducted an investigation to examine whether Brain-PAD has the predictive capability for clinical variables in PD.

Plants

Seed stocks	N/A
Novel plant genotypes	N/A
Authentication	N/A

Magnetic resonance imaging

Experimental design

Design type	Anatomical
Design specifications	we used high resolution T1-weighted MRI data in this study.
Behavioral performance measures	N/A

Acquisition

Imaging type(s)	T1-weighted MRI data
Field strength	1.5-3
Sequence & imaging parameters	The data for this study were collected from various sites participating in the PPMI study. For detailed information about the MRI protocols employed, please refer to: https://www.ppmi-info.org/
Area of acquisition	Whole brain
Diffusion MRI	☐ Used ☒ Not used

Preprocessing

Preprocessing software	CAT12 toolbox (http://www.neuro.uni-jena.de/cat/), as an extension of the Statistical Parametric Mapping (SPM12) software package (https://www.fil.ion.ucl.ac.uk/spm/software/spm12/)
Normalization	Both special and intensity normalization were performed on MRI scans
Normalization template	Spatial normalization was carried out using the Diffeomorphic Anatomical Registration using Exponentiated Lie algebra (DARTEL) algorithm in CAT12. For more details, refer to: https://neuro-jena.github.io/cat/
Noise and artifact removal	Noise removal was performed using the spatial-adaptive Non-Local Means (SANLM) algorithm in CAT12
Volume censoring	N/A

Statistical modeling & inference

Model type and settings	The brain age estimation model was developed using a support vector regression (SVR) algorithm with a linear kernel implemented in MATLAB R2020b (The MathWorks, Natick, MA, USA).
Effect(s) tested	The mean brain-PAD between the hold-out sets was examined using an independent Student's t-test. We used multiple linear regression models to examine whether brain-PAD is able to predict the clinical variables in PD.

Specify type of analysis: ☐ Whole brain ☐ ROI-based ☒ Both

Anatomical location(s): A significant cluster, consisting of 1200 voxels, was predominantly located in the left Parahippocampal Gyrus, extending to Hippocampus and Amygdala.

Statistic type for inference (See Eklund et al. 2016)	The voxel-based morphometry (VBM) technique.
Correction	For each model, we reported the adjusted R2, F-statistic, and p-value. The false discovery rate (FDR) strategy was employed to adjust the p-values.

Models & analysis

n/a	Involved in the study
☒	☐ Functional and/or effective connectivity
☒	☐ Graph analysis
☐	☒ Multivariate modeling or predictive analysis

Multivariate modeling and predictive analysis	We used multiple linear regression models to examine whether brain-PAD is able to predict the clinical variables in PD.
